# Senescence-Associated *Glycine max* (*Gm*)*NAC* Genes: Integration of Natural and Stress-Induced Leaf Senescence

**DOI:** 10.3390/ijms22158287

**Published:** 2021-08-01

**Authors:** Otto Teixeira Fraga, Bruno Paes de Melo, Iana Pedro Silva Quadros, Pedro Augusto Braga Reis, Elizabeth Pacheco Batista Fontes

**Affiliations:** 1 Biochemistry and Molecular Biology Department, Universidade Federal de Viçosa, Viçosa 36570.000, MG, Brazil; otto.netto@ufv.br (O.T.F.); brunopaesdemelo@gmail.com (B.P.d.M.); iana.quadros@ufv.br (I.P.S.Q.); pedroreis@ufv.br (P.A.B.R.); 2 National Institute of Science and Technology in Plant-Pest Interactions, INCTIPP–BIOAGRO, Universidade Federal de Viçosa, Viçosa 36570.000, MG, Brazil; 3 Embrapa Genetic Resources and Biotechnology, Brasília 70770.917, DF, Brazil

**Keywords:** leaf senescence, GmNAC, abiotic stresses, programmed cell death, PCD, NAC transcription factors, soybean, regulatory gene networks

## Abstract

Leaf senescence is a genetically regulated developmental process that can be triggered by a variety of internal and external signals, including hormones and environmental stimuli. Among the senescence-associated genes controlling leaf senescence, the transcriptional factors (TFs) comprise a functional class that is highly active at the onset and during the progression of leaf senescence. The plant-specific NAC (NAM, ATAF, and CUC) TFs are essential for controlling leaf senescence. Several members of Arabidopsis *AtNAC-SAGs* are well characterized as players in elucidated regulatory networks. However, only a few soybean members of this class display well-known functions; knowledge about their regulatory circuits is still rudimentary. Here, we describe the expression profile of soybean *GmNAC-SAGs* upregulated by natural senescence and their functional correlation with putative *AtNAC-SAGs* orthologs. The mechanisms and the regulatory gene networks underlying *GmNAC081-* and *GmNAC030*-positive regulation in leaf senescence are discussed. Furthermore, new insights into the role of *GmNAC065* as a negative senescence regulator are presented, demonstrating extraordinary functional conservation with the Arabidopsis counterpart. Finally, we describe a regulatory circuit which integrates a stress-induced cell death program with developmental leaf senescence via the NRP-NAC-VPE signaling module.

## 1. Introduction

Leaf senescence is a developmentally programmed or environmentally induced process by which cells activate the programmed cell death response (PCD), resulting in the relocation of nutrients towards different organs. Leaf senescence is not an unregulated cell death process initiated by aging; it is rather a sophisticated biochemical and genetic mechanism that controls cell/tissue/organism development through dynamic modulation of gene expression, metabolic reprogramming, and structural changes [1,2,3,4,5,6]. At the cellular level, leaf senescence can be modulated by environmental stimuli, signaling components, metabolism regulators, and phytohormones, although hormones are the major players in the different stages of leaf senescence.

The dismantling of chloroplasts generates the first visible indication of leaf senescence, leaf yellowing. During this process, carbon assimilation is succeeded by the catabolism of chlorophyll, proteins, and lipids; thereby, their metabolized products are relocated to different organs [1,2]. Chlorophyll catabolism occurs in three different cell compartments: chloroplasts, endoplasmic reticulum (ER)/cytosol, and vacuole. Furthermore, this process requires expression and activation of a group of genes related to chlorophyll catabolism (chlorophyll catabolic genes; CCGs) [1]. In the chloroplasts, chlorophyll a is converted to chlorophyll b by chlorophyll b reductase, and the tetrapyrrole ring is then cleaved to generate the fluorescent chlorophyll catabolite (FCC). FCC is exported to the cytosol/ER and then to the vacuole [2]. The chloroplast dismantling steps do not occur simultaneously, as chlorophyll catabolism precedes protein degradation and structural changes [1]. This entire process is tightly regulated by gene expression control and hormone signaling. Thus, several transcription factors have been identified as modulating the expression of CCG genes upon hormone triggering and stress/environmental conditions [4,5,6,7,8,9].

A complex signaling network is triggered during the leaf senescence process; it is activated by aging or environmental factors, but the mechanisms and players that control the whole process are not fully understood. Nevertheless, forward and reverse genetic studies, next-generation sequencing (NGS), and omics technologies have enabled identification of components, signaling molecules, and different genes involved in several steps of the leaf senescence process [3,4,5,6]. To date, the leaf senescence database (LSD) has broadly listed over 5000 SAGs (senescence-associated genes) and 600 mutants from 68 species [7], and the use of these different tools has provided deep knowledge regarding the leaf senescence mechanism.

Hormones play significant roles over a plant’s lifespan; they are involved in distinct developmental and environmental responses. Regarding leaf senescence, tight hormone control and balance allow the correct onset and progress of senescence signaling pathways [8]. Different hormones have specific functions in the activation/repression and control of leaf senescence. Ethylene, abscisic acid (ABA), jasmonic acid (JA), and salicylic acid (SA) accelerate senescence, while auxin, gibberellic acid (GA), and cytokinins act by delaying the process [8,9]. However, it is worth noting that activation/repression or acceleration/delaying of processes by hormones might depend on the organism and the environmental conditions. Moreover, the role of hormones in the modulation of leaf senescence relies on different signaling transducers, transcription factors, and metabolic and structural enzymes [3,10,11,12,13,14]. Over 5000 genes upregulated by senescence have been identified in plants [7], and some of these have been functionally characterized. Among the different classes of SAGs, the transcription factors (TF) play crucial roles in controlling leaf senescence activation, inhibition, and progress. Leaf senescence is regulated through dynamic crosstalk among the different hormone signaling and TF families, including NAC, WRKY, and MYB [15,16,17].

NAC (an acronym for **N**AM, **A**TAF1,2, and **C**UC2) is a plant-specific family of transcription factors, one of the largest found in the plant kingdom. The NAC transcription factors modulate several cell signaling responses and play significant roles in developmental, hormonal, and stress events [5,18,19,20,21]. The NAC proteins display a well-conserved N-terminal NAC domain, comprising the DNA-binding domain and a variable domain at the C-terminus, harboring the transcriptional regulatory region [20,21]. A genome-wide analysis identified 180 NAC genes in the soybean genome, which were clustered into 15 phylogenetic subfamilies [5]. Roughly 40% of the *Glycine max (Gm)NAC* genes were differentially expressed (DE) during the natural leaf senescence process, and the majority of DE genes were upregulated at the initial phase of leaf senescence. This large proportion of senescence-associated NAC genes has also been observed in Arabidopsis and rice [3,22,23]. Several SAG-NACs from Arabidopsis have been functionally characterized and key senescence regulatory networks elucidated [9,24,25,26]. Despite the longstanding interest in crop leaf senescence, few similar studies have been conducted in soybean, although some mechanisms underlying GmNAC-mediated leaf senescence have been partially elucidated.

This review focuses on the roles of senescence-associated *GmNAC* genes in developmentally programmed leaf senescence and their integration with signaling modules of stress-induced leaf senescence. We discuss possible GmNAC-derived senescence regulatory networks and the underlying mechanism of GmNAC-mediated leaf senescence. Furthermore, we highlight the profiles of the senescence-associated *GmNAC* genes under different stress conditions and their possible connections with the leaf senescence process.

## 2. Leaf Senescence-Associated *GmNAC* Genes: Expression Profile and In Silico Analyses

The main feature of leaf senescence is the extensive gene expression reprogramming and subsequent physiological changes, which transform leaves from naturally nutrient-accumulating organs into nutrient-recycling organs [27]. Senescence is typically one of the natural developmental processes; however, it is also triggered by adverse environmental conditions [27,28,29,30,31,32].

**Figure 1:**
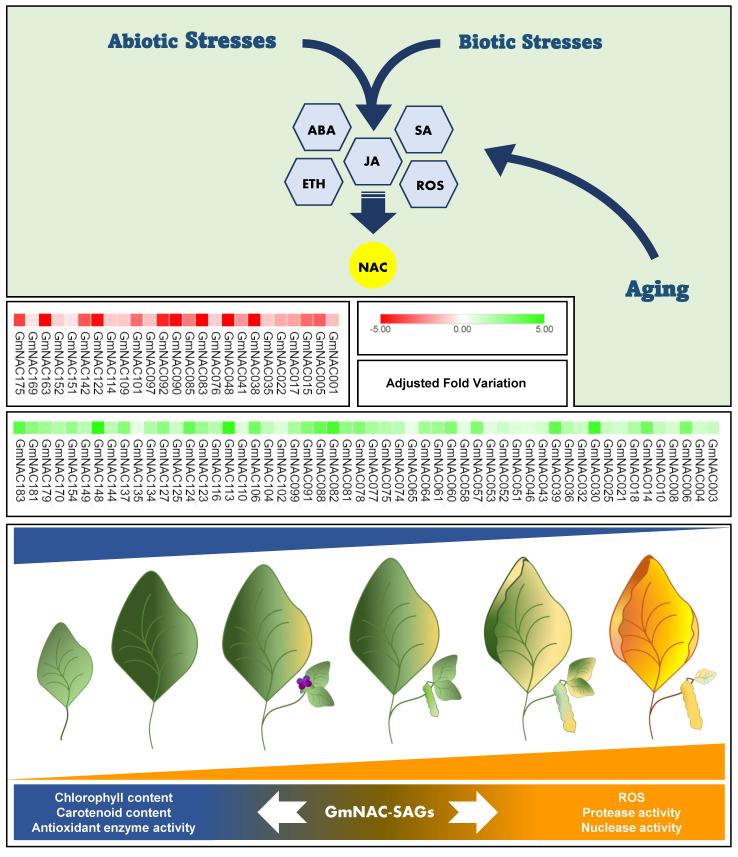
Integrated environmental and developmental senescence mechanisms in soybean and *GmNAC-SAGs* involved in this process. Senescence is a naturally controlled program that culminates in nutrient recycling before plant reproduction. The biological event is complex and finely regulated by several gene networks integrating different TF families, including NAC-TFs. During ongoing senescence, plant metabolism undergoes drastic changes imposed by the extensive gene expression reprogramming coordinated by phytohormones, especially ABA, ETH, JA, and SA. However, these hormones also serve as stress signalers in plants subjected to multiple stresses, overlapping the developmental and stress-responsive mechanisms that converge to programmed cell death (PCD). Conceptually, PCD is classified as developmental PCD (dPCD) and environmental PCD (ePCD), according to the signal’s origin. Since the transcription factors, mainly those from the GmNAC family, are hormone-responsive, dPCD and ePCD might be temporally controlled and partially integrated by *GmNAC-SAGs* responsive to stress. The genes upregulated in senescence are conceptually designated SAGs (senescence-associated genes). Several of these are also stress-responsive and participate in regulatory gene networks that confer stress tolerance or trigger cell death as a stress-evasive program. The *GmNACs* differentially regulated by developmental senescence are shown. The genes upregulated (54) by senescence are shown in green, and the downregulated genes are shown in red. GmNAC TFs may integrate these programs and, as protein nodes, regulate the balance between the plant antioxidant system and aging, the molecular and phenotypical readouts of which are ROS accumulation and chlorophyll and biomolecule breakdown until leaf abscission.

The aging process that culminates senescence is designated developmental programmed cell death (dPCD), whereas senescence as a stress-escape response is designated environmental programmed cell death (ePCD) [31]. dPCD and ePCD share the same molecular basis, resulting in ROS accumulation, chlorophyll loss, and tissue and organ disassembly until abscission [30,33,34], but exhibit different regulatory gene networks [31]. However, several signals for plant physiology adjustment, including the synthesis of phytohormones such as ethylene (ETH), abscisic acid (ABA), jasmonate (JA), auxins (AUX), and salicylic acid (SA), also promote senescence [29]. Regarding the biochemical crosstalk between dPCD and ePCD, some genes conceptually classified as SAGs are also stress-responsive genes (Figure 1).

In *Arabidopsis thaliana*, more than 20% of encoded genes have their expression profiles changed by senescence [35]. A temporal profile of global gene expression variation reveals gene sets that are highly ordered in the control of molecular events associated with leaf senescence [36], and many of these encode transcription factors (TFs) [3,24,29,30,36]. TFs are involved in different developmental processes and act as nodes in signaling processes, connecting signal-sensing and morphophysiological changes [5,24,37,38,39,40]. Therefore, the identification of senescence-associated TFs and dissection of their hormone-controlled regulatory networks represent valuable tools for molecular breeding and the generation of superior crops.

Different TF families participate in the ongoing senescence, mainly the NAC [5,25,28,38,41,42,43,44,45,46,47,48], MYB [13,16], AP2/ERF [49,50], and WRKY [17,51,52,53] families. In Arabidopsis, several regulatory gene networks have already been characterized and the crosstalk regulation between phytohormones and TFs is extensively understood [54]. However, few SAGs have been described in crop plants, including soybean, maize, cotton, sunflower, barley, rice, and wheat, and their regulatory networks in such plant species remain unclear.

Approximately 44% (79 genes) of the GmNAC genes were recently shown to be differentially expressed (DE) at the senescence onset. These DE *GmNACs* predominate in the upregulated (54 genes; 68%) changes over the downregulated (25 genes; 32%) changes (Figure 1) [5]. We classified the *GmNAC* genes upregulated by natural leaf senescence as SAGs. Along with their putative orthologs from *A. thaliana, GmNAC-SAGs* were distributed into the already described NAC subfamilies (Table 1; [5,55]). The most representative subfamilies, harboring the higher number of functionally characterized soybean and Arabidopsis related-genes, are SNAC-A/B, NAM, TERN, TIP, Senu5, and ONAC022. Almost half of the *GmNAC* genes upregulated by natural senescence also respond to at least one type of environmental stress, demonstrating the functional plasticity of NAC TFs. The partial overlapping of GmNAC functions in dPCD and ePCD is also shared by hormone signaling branches controlling these phenomena, as described by well-characterized Arabidopsis gene regulatory networks [24,29,54].

### 2.1. SNAC-A (ATAF) and SNAC-B (NAP) Subfamilies Harbor an Expressive Set of Putative Positive Regulators of Senescence in Soybean

Phylogenetically, the NAC superfamily in soybean is divided into 15 subfamilies, according to sequence and functional conservation between soybean and *A. thaliana* genes [5]. Only two subfamilies, OsNAC8 and ANAC011, do not include a member upregulated by aging (Table 1). SNAC-A (ATAF) is the most represented subfamily in senescence; 90% of the genes are differentially expressed in leaf senescence (10 of 11 members). Within this family, *GmNAC018*, *GmNAC030*, *GmNAC039,* and *GmNAC043* are SAGs displaying stress-responsiveness [5,41] (Table 1). *GmNAC018* and *GmNAC039* are putative paralogs and display similar induction patterns, with expressive upregulation by simulated drought, ER, and biotic stresses [41]. Similarly, *GmNAC030* belongs to a partially overlapped regulatory circuit integrating drought, ER, and biotic stresses with natural senescence in *planta*, and thus is considered a *GmNAC-SAG*. GmNAC030 interacts with GmNAC081 (TERN subfamily), which is involved in both dPCD and ePCD [43,56,57].

**Table 1:** *GmNAC-SAGs* and their putative *AtNAC-SAG* orthologs are responsive to multiple stresses.

Subfamily *	*GmNAC* *	ID	Features **	References	Putative Ortholog in Arabidopsis ***	ID	Features **	References
SNAC-A (ATAF)	*GmNAC018*	Glyma.04G208300	Responsive to drought, ER, and biotic stresses in soybean seedlings, respectively elicited by PEG, tunicamycin, and salicylic acid treatments.	Ferreira et al., 2020 [41]	*ANAC002* *(ATAF1)*	AT1G01720	It is induced by ABA and H_2_O_2_ treatments in Arabidopsis. It belongs to a regulatory network involving ABA-triggered senescence, targeting other SAGs such as *ORE1*.	Wu et al., 2009 [58] Jensen et al., 2013 [59] Garapati et al., 2015 [60] Qiu et al., 2015 [15]
	*GmNAC030*	Glyma.05G195000	Responsive to ER and drought stresses. It belongs to NAC-VPE circuit promoting cell death in natural and stress-induced senescence.	Irsigler et al., 2007 [61] Pinheiro et al., 2009 [18] Mendes et al., 2013 [56]				
	*GmNAC039*	Glyma.06G157400	Phylogenetically grouped with the *GmNAC018* putative paralogue. It displays the same stress responsiveness.	Ferreira et al., 2020 [41]				
	*GmNAC043*	Glyma.06G248900	Upregulated by drought and oxidative stresses. It responds to ABA and air-drying treatments in soybean seedlings.	Melo et al., 2021 [28] Thu et al., 2014 [62] Hussain et al., 2017 [63]	*ANAC055*	AT3G15500	Responsive to drought, salt, ABA, and JA. It integrates a stress-responsive and senescence-promoting circuit together with *ANAC019* and *ANAC072.*	Bu et al., 2008 [64] Hickman et al., 2013 [65] Zhu et al., 2015 [66]
SNAC-B (NAP)	*GmNAC003*	Glyma.01G051300	It responds to ABA treatment in soybean. Highly induced by drought, mainly in roots.	Tran et al., 2009 [67] Quach et al., 2014 [68]	*ANAC029* *(AtNAP)*	AT1G69490	Upregulated by ABA-treatment, drought, and osmotic stresses in Arabidopsis. Also responsive to ethylene.	Guo and Gan, 2006 [48] Jensen et al., 2013 [59]
	*GmNAC010*	Glyma.02G109800	Responsive to dehydration in shoots.	Tran et al., 2009 [67]				
	*GmNAC052*	Glyma.07G229100	Key player in cold responses and flowering-time coordination.	Hussain et al., 2017 [63]				
	*GmNAC148*	Glyma.20G033300	Drought-responsive gene.	Hussain et al., 2017 [63]				
	*GmNAC006*	Glyma.02G070000	Highly responsive to drought in the sensitive soybean cultivar MDT720.	Thu et al., 2014 [62]	*ANAC047*	AT3G04070	Responsive to salt and osmotic stress in Arabidopsis. Also responsive to bacterial infection.	Mito et al., 2010 [69] Shaik and Ramakrishna, 2013 [70]
	*GmNAC127*	Glyma.16G151500	-	-				
	*GmNAC124*	Glyma.16G043200	Responsive to abiotic stresses, conferring salt tolerance in transgenic soybean hairy-roots.	Hao et al., 2011 [71]				
	*GmNAC181*	Glyma.19G108800	Responsive to multiple stresses and plant hormones. It confers salt tolerance in transgenic plants. Regulatory gene network analyses suggest it regulates DREB1A and other stress-related genes.	Hao et al., 2011 [71]				
	*GmNAC091*	Glyma.12G221400	-	-	*ANAC056*	AT3G15510	Downregulated by ABA treatment.	Kleinow et al., 2009 [72] Aslam et al., 2012 [73]
	*GmNAC102*	Glyma.13G280000	Responsive to dehydration in soybean roots during vegetative and reproductive stages.	Le et al., 2012 [74]				
	*GmNAC099*	Glyma.13G243200	-	-	*ANAC025*	AT1G61110	-	-
	*GmNAC113*	Glyma.15G070300	-	-				
NAM	*GmNAC149*	Glyma.20G172100	Slightly induced by severe drought.	Carvalho et al., 2014 [75] Melo et al., 2021 [28]	*ANAC017*	AT1G34190	Not responsive to classical abiotic stresses. Associated with mitochondrial stresses and consequent H_2_O_2_ accumulation.	Ng et al., 2013 [76] Meng et al., 2019 [77]
	*GmNAC074*	Glyma.10G219600						
	*GmNAC182*	Glyma.19G165600	-	-	*ANAC074*	AT4G28530	-	-
	*GmNAC058*	Glyma.08G156500	Upregulated by persistent water stress conditions.	Carvalho et al., 2014 [75] Silva et al., 2015 [78]	*ANAC103*	AT5G64060	-	-
	*GmNAC061*	Glyma.08G173400	-	-	*ANAC022*	AT1G56010.2	-	-
	*GmNAC125*	Glyma.16G051800	-	-	*ANAC083* *(VNI2)*	AT5G13180	Upregulated by salt and ABA treatments. Negative regulator of natural senescence in Arabidopsis.	Yang et al., 2011 [26]
	*GmNAC123*	Glyma.16G042900	Responsive to drought in late vegetative stages.	Le et al., 2012 [74]	*ANAC087*	AT5G18270	-	-
	*GmNAC144*	Glyma.19G109100	-	-				
TERN	*GmNAC077*	Glyma.11G096600	Downregulated by severe drought.	Carvalho et al., 2014 [75] Melo et al., 2021 [28]	*ANAC036*	AT2G17040	-	-
	*GmNAC081*	Glyma.12G022700	Upregulated by drought and ER stresses. Transgenic plants overexpressing GmNAC081 display accentuated drought sensitivity and accelerated senescence phenotypes.	Irsigler et al., 2007 [61] Pinheiro et al., 2009 [18] Faria et al., 2011 [19] Pimenta et al., 2016 [43] Ferreira et al., 2020 [41]				
	*GmNAC078*	Glyma.11G182000	-	-	*ANAC090*	AT5G22380	Expression remains unaltered during stress responses but the protein acts as a negative regulator of ROS and SA pathways.	Kim et al., 2018 [42]
	*GmNAC082*	Glyma.12G091200	Highly responsive to mild–severe and severe drought stresses in the tolerant Jindou soybean cultivars.	Hussain et al., 2017 [63]				
	*GmNAC088*	Glyma.12G186200	-	-				
	*GmNAC104*	Glyma.13G315300	-	-				
	*GmNAC014*	Glyma.03G197900	-	-				
	*GmNAC053*	Glyma.07G271100	Key player in cold responses and flowering-time coordination.	Hussain et al., 2017 [63]	*ANAC035*	AT2G02450	-	-
ONAC022	*GmNAC060*	Glyma.08G169400	-	-	-	-	-	-
	*GmNAC116*	Glyma.15G257700	-	-	-	AT3G12910	-	-
	*GmNAC106*	Glyma.14G030700	Negatively regulated by bleomycin (cell death inducer) treatment.	Melo et al., 2021 [28]	*ANAC042* *(JUB1)*	AT2G43000	Responsive to H_2_O_2_ accumulation. Negative regulator of natural senescence. Also responsive to flagellin-PAMP.	Wu et al., 2012 [79] Saga et al., 2012 [80]
	*GmNAC154*	Glyma.02G284300	Slightly induced by bleomycin treatment.	Melo et al., 2021 [28]				
	*GmNAC064*	Glyma.08G307100	-	-				
	*GmNAC137*	Glyma.18G110700	-	-				
	*GmNAC134*	Glyma.17G185000	-	-	*ANAC083* *(VNI2)*	AT5G13180	Upregulated by salt and ABA treatments. Negative regulator of natural senescence in Arabidopsis.	Yang et al., 2011 [26]
ANAC063	*GmNAC004*	Glyma.01G088200	Upregulated by drought stress in soybean. Arabidopsis ectopically expressing the soybean gene displays a hallmarked lateral root formation under drought.	Quach et al., 2014 [68] Hussain et al., 2017 [63]	*ANAC008*	AT1G25580	-	-
	*GmNAC008*	Glyma.02G100200	-	-				
	*GmNAC170*	Glyma.10G204700	-	-				
	*GmNAC025*	Glyma.05G002700	-	-	*ANAC044*	AT3G01600	Responsive to genotoxic stresses: bleomycin, hydroxyurea, mitomycin C, and methanesulfonate.	Takahashi et al., 2019 [81]
TIP	*GmNAC021*	Glyma.04G226700	Highly responsive to mild–severe and severe drought stresses in the tolerant Jindou soybean cultivars.	Hussain et al., 2017 [63]	*NTL9*	AT4G35580	Responsive to osmotic stress.	Yoon et al., 2008 [82]
	*GmNAC110*	Glyma.14G189300	*pGmNAC110* harbors a UPR-cis regulatory element, suggesting some responsiveness in ER stress.	Sun et al., 2013 [83] Silva et al., 2015 [78]				
	*GmNAC036*	Glyma.06G138100	Predicted *GmNAC062* ortholog. Its promoter harbors a UPR-cis regulatory element.		*ANAC090*	AT5G22380	Expression remains unaltered during stress responses but the protein acts as a negative regulator of ROS and SA pathways.	Kim et al., 2018 [42]
ANAC001	*GmNAC051*	Glyma.07G201800	-	-	*ANAC073*	AT4G28500	-	-
	*GmNAC032*	Glyma.05G225100	-	-	*ANAC099*	AT5G56620	-	-
Senu5	*GmNAC065*	Glyma.08G360200	Differentially responsive to PEG, tunicamycin, and salicylic acid treatments in soybean. Highly responsive to ABA. Ectopically expressing Arabidopsis transgenic lines display delayed senescence and an enhanced antioxidant system when subjected to abiotic and biotic stresses. *GmNAC065* is also upregulated in drought-tolerant soybean plants.	Hussain et al., 2017 [63] Melo et al., 2018 [5] Melo et al., 2021 [28]	*ANAC083* *(VNI2)*	AT5G13180	Upregulated by salt and ABA treatments. Negative regulator of natural senescence in Arabidopsis.	Yang et al., 2011 [26]
	*GmNAC179*	Glyma.18G301500	Predicted *GmNAC065* paralog. It displays a similar stress-induction pattern. Slightly induced by bleomycin treatment.	Melo et al., 2021 [28]				
VND-NAC	*GmNAC075*	Glyma.11G030600	-	-	*ANAC007*	AT1G12260	-	-
Unnamed Group	*GmNAC135*	Glyma.17G240700	-	-	*ANAC011*	AT1G32510	Responsive to wounding.	Matsuoka et al., 2021 [84]
	*GmNAC057*	Glyma.08G075300	Upregulated by water stress. The drought-susceptible cultivar MDT777-2 displays higher gene expression than tolerant cultivars. In reproductive stages, under drought, the gene appears to be downregulated.	Le et al., 2012 [74] Thu et al., 2014 [62]	*ANAC104*	AT5G64530	-	-
	*GmNAC046*	Glyma.07G048000	Cold-responsiveness. Possibly associated with the control of flowering.	Hussain et al., 2017 [63]	*NTL9*	AT4G35580	Responsive to osmotic stress.	Yoon et al., 2008 [82]

(*) According to the last investigation of GmNAC superfamily in the soybean genome (Melo et al., 2018) [5]. (**) Reports associating NAC TFs with different stresses. (***) According to deduced amino acid sequence homology, defined by Phytozome and Soybase BALSTp in-house algorithms (https://phytozome.jgi.doe.gov/pz/portal.html#!info?alias=Org_Gmax and https://www.soybase.org/GlycineBlastPages/).

Finally, *GmNAC043* is also referred to as a putative *GmNAC-SAG*, as previously reported by Melo et al. in 2021 [28]. *GmNAC043* is responsive to different stresses, such as drought, oxidative stress, and insect and fungus attack, and is slightly upregulated by natural senescence (Table 1). Furthermore, *GmNAC043* (*ANAC055* ortholog) is significantly induced by bleomycin treatment in soybean, reinforcing its role as a gene involved in programmed cell death control [28].

The putative orthologs of SNAC-A (ATAF) soybean genes identified in Arabidopsis belong to very well-described regulatory circuits integrating multiple stress responses and senescence (Table 1). *ATAF1* (*ANAC002*) integrates ABA-hormone signaling and senescence by simultaneously suppressing the expression of photosynthesis-associated genes and stimulating the expression of other SAGs [24,85]. Since it is a hormone-responsive TF, several stressful conditions that culminate in ABA and ROS production activate *ATAF1* expression [15,58,59,60]. Additionally, *ATAF1* represents a classical *AtSAG* since it promotes dark and developmental senescence, which is remarkably delayed in *ataf1* mutants [60].

The *GLK1* and *ORE1* promoters harbor *cis*-acting elements directly targeted by ATAF1. Accordingly, ATAF1 upregulates the expression of *ORE1*, which shares a similar expression pattern as *ATAF1* [54]. Conversely, ATAF1 downregulates the expression of chloroplast-maintenance genes including *GLK1*, which delays senescence when overexpressed in plants. Interestingly, ORE1 directly interacts with GLK1 to inhibit its activity [86]. Therefore, *ATAF1* is activated by ABA during natural and stress-induced senescence and in turn activates the expression of ORE1 which, together with ATAF1, regulates the chloroplast-maintenance function of GLK1, providing a feedforward loop to promote photosynthesis decay and leaf yellowing leading up to abscission. Not surprisingly, ATAF1 targets genes involved in ABA biosynthesis and transport, *NCED3* and *ABCG40*, respectively, which are essential for ABA signaling [59]. Despite the high structural conservation of SNAC-A (ATAF)-SAGs, a soybean ortholog of ATAF1, GmNAC030, forms a heterodimer with GmNAC081 to repress the expression of *ABCG40* and regulators of ABA signaling [41]. These results indicate that the underlying mechanism of NAC-SAG-mediated senescence may differ across species.

Additionally, in the SNAC-A (ATAF) subfamily, GmNAC085 forms a divergent and distant clade with GmNAC101, GmNAC092, and GmNAC043, which differ from their Arabidopsis orthologs in expression profiles in response to natural leaf senescence [5,28]. With the exception of GmNAC043-SAG, which shares a similar expression profile with the orthologs in Arabidopsis ANAC072, ANAC019, and ANAC055, the other soybean members of this clade are downregulated by developmentally programmed leaf senescence [5,28,65]. Despite the divergent expression profile, there is a lack of information about the molecular functions of these GmNACs and the redundant functional aspects of their paralogs remains unclear. Ectopic expression of GmNAC085 in *Nicotiana benthamiana* leaves leads to classic symptoms of senescence, including chlorophyll loss, H_2_O_2_ accumulation, and leaf yellowing [5]. Similar phenotypes are also observed in transgenic Arabidopsis lines expressing the soybean gene. In addition, these plants display an imbalanced enzymatic and metabolic antioxidant system, with low expression and activity of superoxide dismutase (SOD), catalase (CAT), and ascorbate peroxidase (APX), associated with low concentrations of soluble sugars, carotenoids, and anthocyanins [28]. Although *GmNAC085* is not considered a conceptual SAG, as it is downregulated during senescence, these findings strongly suggest that it may be associated with the control of ePCD in soybean. Consistent with a role in ePCD, co-expression analyses revealed that *GmNAC085* positively correlates with ABA-responsive genes, genes involved in aromatic amino acid metabolism, and genes associated with protein and metabolite breakdown, mainly the subunits of proteasome complex, endopeptidases, and peroxidases [28].

The SNAC-B (NAP) subfamily also harbors an expressive number of senescence-upregulated genes. Among 23 genes, 12 genes are induced by the onset of senescence, representing almost 50% of soybean SNAC-B (NAP) genes. *GmNAC006*, *GmNAC010*, *GmNAC102*, *GmNAC148*, and *GmNAC181* are responsive to drought [62,63,67,68,71,74] (Table 1). *GmNAC052* is upregulated by cold stimuli and may control the ongoing senescence in flowers [63]. Likewise, *GmNAC046* (unnamed group) is positively regulated by senescence. Displaying a similar expression pattern as *GmNAC181*, *GmNAC124* might be involved in salt-stress responses and soybean hairy-root development; overexpression of these GmNAC-SAGs confers tolerance to osmotic stresses [71]. *GmNAC181*-overexpressing Arabidopsis lines (previously referred to as *GmNAC11*) show a more than 60% survival rate after recovery from 15 days exposure to salt stress. The *GmNAC181-*mediated increases in osmotic stress tolerance have been associated with the DNA-binding activity of GmNAC181, which directly targets *cis*-elements in the DREB-1A promoter. Gene expression analyses in *GmNAC085*-overexpressing lines revealed enhanced expression of *DREB-1A*, *ERD11*, *COR15A*, *ERF5*, *RAB18,* and *KAT2*, frequently associated with multiple stress-tolerance phenotypes [71].

The putative orthologs from the SNAC-B (NAP) subfamily in Arabidopsis also respond to multiple stresses (Table 1). *ANAC047* (*GmNAC006*, *GmNAC124*, *GmNAC127,* and *GmNAC181*) respond to salt- and mannitol-induced osmotic stresses with late to intermediate kinetics, with higher transcript accumulation after 12 h of treatment [69]. Transgenic *A. thaliana* lines expressing the chimeric repressor ANAC047-SRDX display high salt tolerance, implicating *ANAC047* as a positive regulator of stress-induced senescence [69]. Interestingly, *ANAC047* also responds to bacterial infection [70]. Bacterial and fungal infections trigger SA-responsive pathways, stimulating oxidative burst and leading to cell death in hypersensitive reactions (HR) [87]. Lipid peroxides accumulate during HR because of ROS production and membrane degeneration, the most common features during ePCD [88].

Another *AtNAC* ortholog from the SNAC-B (NAP), *ANAC029 (AtNAP)*, integrates a senescence regulatory network with *ANAC055* and *ANAC072*, SNAC-A subfamily gene members [55,89] (reviewed by [29,54]). These genes are phylogenetically related and display the same expression patterns induced by ABA and different abiotic stresses [38,55,90] (Table 1). The senescence circuit integrated by ANAC019 and ANAC055 encompasses components of the EIN2 (ETHYLENE INSENSITIVE 2) senescence-regulatory pathway [12,91]: ANAC019 and ANAC055 are direct targets of EIN3 TF, a downstream component of the *EIN2* cascade in ETH-mediated signaling [92]. EIN3 can bind *cis*-elements in the promoter of several *AtNAC-SAGs*, including *ANAC019*, *ANAC055,* and *ANAC059 (ORS1)* [93]. For *ANAC072*, the activation pathway diverges in some aspects from its closely related genes. The promoter region of *ANAC072* harbors *cis*-elements recognized by CBF (C-REPEAT/DEHYDRATION RESPONSIVE BINDING FACTORS), which is frequently associated with senescence progression [65,94].

### 2.2. Putative GmNAC-SAGs also Share Structural Conservation with Negative Regulators of Senescence Progression from Arabidopsis

Few soybean and Arabidopsis NAC TFs from the NAM subfamily have been associated with stress-responsive and/or senescence-regulatory gene networks. A total of 16 of 42 *GmNAC* genes (38%) of the NAM members are upregulated in soybean during natural senescence. The NAM subfamily encompasses gene members typically related to growth and developmental processes [5]; however, the putative *GmNAC125* ortholog, *ANAC083 (VNI2)*, belongs to a negative regulatory cascade of senescence in Arabidopsis [26] (Table 1). *ANAC083* integrates ABA signaling and controls xylem vessel specification [26,95]. *ANAC083-*overexpressing plants are remarkably tolerant to salt and drought, as well as displaying a delayed senescence phenotype. The higher expression levels of *ANAC083* lead to a significantly higher expression of *COR/RD* genes [26], shared with the AREB-1 drought-tolerance pathway. Multiple physiological events upregulate *RD29A*, *RD29B*, *RD22,* and *RD20* genes as plants try to cope with long-standing adverse conditions before activating ePCD [96,97,98,99,100], culminating in improved plant performance under adverse conditions [101]. Similarly, *GmNAC065* (Senu5), a putative *ANAC083* ortholog, also acts as a negative regulator of natural and stress-induced senescence [28].

Curiously, all of the ONAC022 subfamily members in soybean (except *GmNAC006* and *GmNAC116*, for which putative orthologous genes have not been identified; Table 1) are phylogenetically related to negative regulators of natural and stress-induced senescence in Arabidopsis. *GmNAC134* is a putative ortholog of *ANAC083. GmNAC064*, *GmNAC137*, *GmNAC106,* and *GmNAC154* are phylogenetically grouped with *ANAC042 (JUB1)*. *GmNAC106* and *GmNAC154* are considered putative *GmNAC-SAGs* and are involved in aging but not in environmental stresses, consistent with their slight induction in bleomycin-treated soybean seedlings [28].

Like *ANAC083 (VNI2)*, *ANAC042 (JUB1)* is a negative regulator of senescence and increases plant longevity in Arabidopsis. Its expression is promptly induced by H_2_O_2_ accumulation, and overexpression of *ANAC042* (*JUB1*) confers tolerance to abiotic stresses, including heat and salt, and delays natural senescence. The opposite effect is observed in *jub1* mutants [79]. As described for the soybean TF GmNAC181, JUB1 directly activates DREB-2A, which targets RD29A from the COR/RD stress evading circuit [26,71,102]. Accordingly, DREB-2A activates the *Hsf-A3* gene [103], which takes part in a feedforward regulatory loop that upregulates genes of heat-shock proteins (Hsps) and ROS-scavenging-related proteins [104,105,106,107].

A large proportion of the GmNAC-SAGs respond to an array of biotic and abiotic stresses (Table 1), but functional information is restricted to GmNAC030, GmNAC065, GmNAC181, and GmNAC118. Remarkably, these characterized GmNAC-SAGs are functionally similar to the Arabidopsis orthologs ATAF1, ANAC083, ANAC036, and ANAC047, respectively. Table 1, which compiles information about structural conservation, phylogenetic relationships, and expression profiles of GmNAC-SAGs and AtNAC-SAGs, provides reliable information to rationalize functional studies of GmNAC-SAGs.

### 2.3. GmNAC-SAGs as Positive and Negative Regulators of Leaf Senescence

Despite the large fraction of *GmNACs* upregulated during leaf senescence, functional information is restricted to a few members of the *GmNAC-SAG* class. One such example is *GmNAC081* (previously designated *GmNAC6*), one of the first *GmNACs* to be identified and cloned [108], which has been shown to be induced by abiotic stress [61] and cell death inducers, is repressed by senescence inhibitors [18], and is classified as a TERN subfamily member [18,108]. It induces necrotic lesions when expressed in tobacco leaves [18] with hallmarks of PCD [19,109]. Full-length GmNAC081 does not transactivate transcription in yeast, yet a truncated GmNAC081 protein, expressing the carboxyl region alone in yeast, functions as a transactivator domain [18,67]. The lack of transactivation activity of GmNAC081 may be due to inhibitory interactions between the C-terminal transactivator domain and the N-terminal NAC DNA-binding domain, which are supposed to be relieved by expressing the C-terminal region alone. The negative interactions between GmNAC081 domains may also be relieved by interactions with other TFs upon heterodimer formation. Accordingly, GmNAC081 has been shown to interact in yeast and in plants with another GmNAC-SAG, GmNAC030, which exhibits transactivation activity in yeast and induces PCD in GmNAC030-expressing soybean protoplasts [56]. The GmNAC081-GmNAC030 heterodimer enhances the transcriptional regulation of shared target promoters, indicating that heterodimerization is required to fully regulate gene expression. Furthermore, as partners, GmNAC081 and GmNAC030 are coordinately regulated in response to multiple environmental and developmental stimuli [43,56]. Among the GmNAC081–GmNAC030 target genes, the vacuolar processing enzyme (VPE) may be responsible for executing a cell death program induced by activation of these TFs [43,56,75]. VPE is a caspase-like 1 protein that executes plant-specific cell death via vacuole collapse, resulting in extensive biomolecule degradation [110].

*GmNAC08*1 has also been functionally characterized as a downstream component of the DCD/NRP-mediated cell death response [19,56] and as a positive regulator of leaf senescence [5,28,41,43]. Transcript expression analysis and *GmNAC081* promoter-GUS assay indicated that GmNAC081 transcriptional control is correlated with leaf senescence events [41,43]. Both soybean and tobacco leaves display low GmNAC081 promoter activity and expression at the early developmental stages, while GmNAC081 transcript level and GUS activity increase in the late stages. Similarly, *GmNAC081*-overexpressing plants show accelerated senescence, a phenotype associated with accelerated leaf yellowing, decreased photosynthesis rate, and enhanced upregulation of SAG marker genes. Additionally, the expression of GmNAC081 target genes encoding cell-death-inducing hydrolytic enzymes are coordinately upregulated with GmNAC081 expression in the late stages of development [43,56]. Conversely, silencing of *GmNAC081* by VIGS (virus-induced gene silencing) in soybean plants causes a delay in leaf senescence. Leaf yellowing and expression of the GmNAC081 target genes during leaf senescence are significantly lower in silenced leaves than in wild-type leaves [43].

Consistent with the role of *GmNA0C81* in senescence, a subset of DEGs (differentially expressed genes) in natural leaf senescence in wild-type leaves is significantly over-represented at the early developmental stage in GmNAC081-overexpressing soybean lines [41]. Therefore, GmNAC081 regulates leaf senescence by modulating the expression of senescence-, cell-death-, and hormone-signaling-related genes [41,43]. GmNAC081 functions either as a transcriptional activator or repressor of TGTG(T/G/C) cis-element-containing target promoters [56]. Analysis of the GmNAC081-induced transcriptome in leaves at early developmental stages uncovered both downregulated senescence-inhibiting and upregulated senescence-promoting genes harboring GmNAC081-binding sites on their promoters [41]. As a positive regulator of leaf senescence, GmNAC081 activates the expression of senescence-inducing genes and negatively modulates the expression of senescence-suppressing genes.

In contrast to GmNAC081 and GmNAC030, positive regulators of leaf senescence, GmNAC065 negatively regulates natural and stress-induced senescence [74]. *GmNAC065* was first identified as a drought-induced gene in soybean leaves at the late vegetative V6 stage and reproductive R2 stage [74]. In contrast, an analysis of drought-responsive genes in soybean leaves at the V3 developmental stage revealed that *GmNAC065* was strongly downregulated by gradually declining the leaf water potential to a maximum stress of −2.0 MPa [75]. The apparent contradiction between these earlier studies may be due to drought induction at different developmental stages, suggesting that specific pathways regulating the drought response might operate in different stages of plant development. As a negative regulator of leaf senescence, the induction of *GmNAC065* by drought may modulate leaf longevity and the stay-green phenotype under stress conditions. Accordingly, a comparison of *GmNAC065* expression in drought-tolerant and drought-sensitive soybean genotypes demonstrated that *GmNAC065* is induced by drought to a greater extent in drought-resistant genotypes, suggesting the potential of *GmNAC065* as a target for molecular breeding or genetic engineering of drought tolerance in soybean [63].

*GmNAC065* has also been demonstrated to be widely responsive to biotic and abiotic stimuli in soybean seedlings treated with the osmotic stress inducer PEG, the ER stress inducer tunicamycin, and the pathogen defense hormone salicylic acid (SA), displaying early induction kinetics [5]. Nevertheless, ectopic expression of GmNAC065 in *N. benthamiana* leaves induces weaker cell death phenotypes compared to those displayed by the stress-induced GmNAC085 and the senescence-associated GmNAC081 expression [5].

*GmNAC065* and its paralog *GmNAC179* are phylogenetically most related to the negative regulator of senescence in Arabidopsis *ANAC083 (VNI2)*, and together they form a closely related branch with another negative regulator of senescence, *ANAC042 (JUB1)*, and its putative orthologous SAGs in soybean, *GmNAC106* and *GmNAC154* [5,28]. These findings support a negative role of *GmNAC065* in leaf senescence. Accordingly, in silico co-expression analyses revealed a strong positive correlation of *GmNAC065* expression profile with stress-sensing- and signal-transduction-associated genes, besides those associated with cell survival under multiple stresses, including LEA proteins, β-carotene hydroxylase, acyl-CoA oxidase, and ubiquinol oxidase. Not surprisingly, these proteins and enzymes are frequently associated with ROS-avoiding mechanisms, photosynthetic apparatus preservation, jasmonate-mediated tolerance to biotic stresses, and drought tolerance [111,112,113]. Overall, *GmNAC065* is co-expressed with genes involved in plant maintenance and redox homeostasis, possibly sharing a redox-balanced environment that results in senescence delay since low ROS levels activate signal pathways that mediate stress escape. However, at higher concentrations, ROS induces PCD [88,114].

Further evidence that *GmNAC065* negatively regulates leaf senescence was provided by overexpressing *GmNAC065* in Arabidopsis [28]. The Arabidopsis transgenic lines ectopically expressing *GmNAC065* exhibited stunted growth compared to wild-type plants during the vegetative stage and a decelerated progression throughout the reproductive stage with delayed leaf senescence, a phenotype similar to that displayed by *ANAC083 (VNI2)*-overexpressing lines [26,28]. Additionally, *GmNAC065*-overexpressing transgenic plants exhibited enhanced expression of *COR/RD* genes, the targets of ANAC083 (VNI2), the Arabidopsis *GmNAC065* ortholog [26], as well as reduced expression of genes associated with the catabolism of proteins and pigments [28]. Furthermore, in the *GmNAC065*-overexpressing lines, the expression and activity of the SOD, CAT, and APX, components of the antioxidant system were increased compared to the wild type. Moreover, the transgenic lines displayed higher contents of soluble sugar, carotenoids, and anthocyanin, as parts of the nonenzymatic plant antioxidant system acting in water retention, UV protection, and ROS scavenging, respectively [115]. Finally, the extent of stress-induced cell death in leaves and roots of *GmNAC065*-overexpressing lines was lower compared to the wild-type control. Remarkably, *GmNAC065* overexpression in Arabidopsis affected the expression of different SAGs and their downstream targets, predominantly as downregulation. *AtNAP*, *ATAF1*, *ORE1*, *ANAC016*, *ANAC019*, and *ANAC055* were slightly induced by *GmNAC065* overexpression, and the downstream targets *NYC1 (NONYELLOW COLORING 1)*, *SINA1 (SEVEN IN ABSENTIA)*, *BFN1 (BIFUNCTIONA NUCLEASE1)*, *BSMT1 (S-ADENOSYLMETHIONINE-DEPENDENT METHYL-TRANSFERASE)*, *GLK1 (GOLDEN2-LIKE 1)*, and *SAG113* were suppressed.

Collectively, these findings suggest that GmNAC065 integrates a negative senescence-regulatory pathway via activation of *RD* genes (upregulated in GmNAC065-overexpressing lines) and, consequently, downregulation of *AtNAC-SAGs* and their downstream targets, executors of stress-triggered programmed cell death. Besides the robust enzymatic and nonenzymatic antioxidant systems and the attenuated senescence phenotype under multiple stresses and normal development, the functional characterization of *GmNAC065* provides new insights into SAGs in soybean and highlights the relevance of these genes as hotspots for biotechnological soybean breeding.

## 3. A Regulatory Circuit Integrating Stress-Induced with Natural Leaf Senescence

Both developmental and stress stimuli have been shown to trigger leaf senescence. Our knowledge about the crosstalk between stress-induced and developmentally programmed leaf senescence has advanced considerably through reverse/forward genetics, multiple omics-based technologies, and expression profiling studies under different stress conditions in Arabidopsis [9]. A consensus theme that has emerged from studies of regulatory networks controlling leaf senescence is the existence of a signaling module in plants integrating the transduction of environmental and developmental signals at the onset and progression of leaf senescence. In soybean, the developmental cell death (DCD) domain-containing asparagine-rich protein (NRP)-mediated cell death response has been characterized as a regulatory circuit that integrates stress-induced with developmentally programmed leaf senescence [57,116].

**Figure 2:**
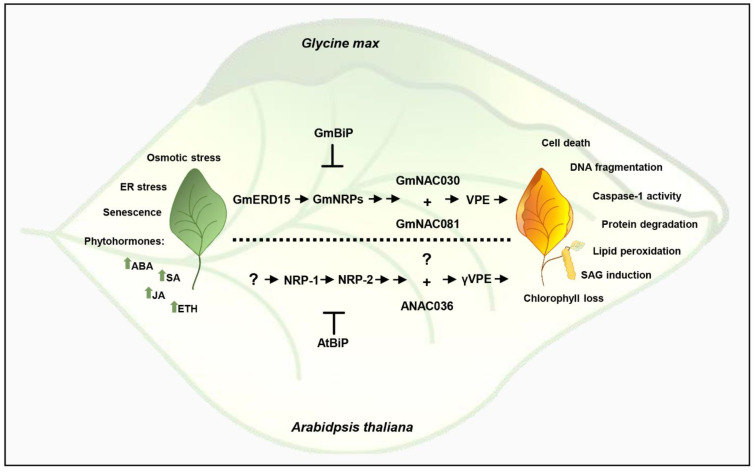
Integration of senescence signals and stress signals in the DCD/NRP-mediated cell death response. Leaf senescence, ER stress, and osmotic stress induce the expression of GmERD15, which upregulates GmNRPs to initiate a signaling cascade that culminates in the induction of GmNAC030 and GmNAC081 expression. The NAC transcription factors form a heterodimer to fully induce VPE promoter activation, leading to VPE expression and subsequent execution of a cell death program. The phytohormones positively regulate the signaling pathway, and the ER-resident molecular chaperone BiP acts as a negative regulator of cell death by modulating the expression and activity of the cell death pathway components. This DCD/NRP-mediated cell death signaling is conserved in other plant species, and the Arabidopsis orthologs are shown at the bottom of the figure.

The DCD/NRP-mediated cell death signaling pathway, which has been uncovered in soybean, transduces a cell death signal derived from multiple stress conditions and late stages of leaf development [116]. The first components of this cell death pathway identified were DCD/NRPs, designated GmNRP-A and GmNRP-B, which gave the pathway its name (NRP signaling; Figure 2). NRPs were isolated in a wide-genome transcriptional screening for commonly induced genes as candidate regulatory components that integrate the ER and osmotic stress responses [61]. GmNRP-A and GmNRP-B belong to the subgroup I of plant-specific DCD-containing proteins [117]. They share a highly conserved C-terminal DCD domain in addition to a high content of asparagine residues at their more divergent N-terminus. The screening for genes synergistically induced by combined stress conditions also identified a NAC transcription factor, GmNA0C81, which was later characterized as a downstream component of the DCD/NRP-mediated cell death pathway [19,61]. The overexpression of NRPs or GmNAC081 in plants induces cell death and apoptotic-like phenotypes such as foliar necrotic lesions, DNA fragmentation, caspase activity, chlorophyll loss, and lipid peroxidation [19,109,118].

Further progress in deciphering the NRP signaling identified GmNAC030 as the GmNAC081 cellular partner [56] (Figure 2). GmNAC030 cooperates with GmNAC081 to bind to target promoters, and the transcriptional complex either induces the expression of activators or represses the expression of inhibitors of the cell death program [41,56]. Among the target hydrolytic enzymes, the expression of VPE is fully induced by the GmNAC081–GmNAC030 heterodimer. The vacuole-localized cysteine protease VPE has caspase-1 activity and is synthesized as an inactive proprotein precursor [119]. The precursor is self-catalytically converted into the active mature form, which in turn mediates the activation of vacuolar enzymes that degrade the vacuolar membrane, resulting in vacuolar-collapse-mediated cell death [120]. Therefore, the NRP/GmNAC081–GmNAC030/VPE signaling module transduces a stress-induced cell death signal into a PCD response.

The multiple-stress responsive gene GmERD15 is the most upstream component of the DCD/NRP-mediated cell death signaling that has been identified to date. ERD15 is a small acidic and hydrophilic protein that belongs to the PAM2-domain-containing protein family, induced by diverse biotic and abiotic stress such as light, cold, high salinity, and drought, osmotic, and ER stress [121,122,123]. Its crucial role as a negative regulator of the abscisic acid (ABA)-mediated response and a positive regulator of the salicylic acid (SA)-dependent defense pathway is consistent with its plasticity in response to multiple environmental stressors [124]. GmERD15 was isolated via its capacity to associate stably with the GmNRP-B promoter in vivo and in vitro to induce the *GmNRP-B* expression [125]. Although ERD15 from Arabidopsis does not bind to NRP promoters, the Arabidopsis orthologs of the NRP/GmNAC081–GmNAC030/VPE signaling module components have been shown to transduce a cell death signal in Arabidopsis with similar regulatory mechanisms [44,125]. The functional orthologs of the soybean pathway components were identified in Arabidopsis as *AtNRP-1* and *AtNRP-2* (GmNRPs), *ANAC036* (*GmNAC081*), *ATAF2*/ATAF1 (*GmNAC030*), and *gVPE;* they have been shown to be induced by ER and osmotic stress and to induce cell death when transiently expressed in *N. benthamiana* leaves. Importantly, knockout lines of the selected Arabidopsis orthologs display enhanced tolerance to ER stress-mediated cell death [44,109]. Conserved sequences of the pathway components are present in several plant genomes; they represent a plant-specific signaling pathway widely distributed in the plant kingdom.

Besides being synergistically activated by multiple stresses conditions, the DCD/NRP-mediated programmed cell death pathway is also associated with developmentally programmed leaf senescence. The expression of cell death pathway components NRP-A, NRP-B, GmNAC081, GmNAC0030, and VPE is associated with the onset and progression of leaf senescence. Furthermore, caspase-1 activity of the executioner of the DCD/NRP-mediated cell death VPE is highly increased in leaf senescence [5,43,75]. The transient expression in plants of soybean and Arabidopsis components of the cell death pathway induces a cell death response with hallmarks of leaf senescence, including induction of caspase-1-like activity and DNA fragmentation, chlorophyll loss, protein degradation, enhanced lipid peroxidation, and the induction of senescence-associated marker genes [44,56,109,118]. Furthermore, the phytohormones ABA, jasmonic acid (JA), and salicylic acid (SA), which positively regulate senescence, also induce the expression of DCD/NRP-mediated cell death pathway components [43]. More strictly, GmNAC081 overexpression accelerates leaf senescence, associated with enhanced chlorophyll degradation, faster photosynthetic decay, and higher expression of hydrolytic-enzyme-encoding GmNAC081/GmNAC030 target genes including VPE, the executioner of programmed cell death [43]. Unlike the accelerated leaf senescence phenotype displayed by the GmNAC081-overexpressing lines, VIGS-mediated suppression of GmNAC081 expression delays leaf senescence and decreases the expression of GmNAC081 direct target genes, including VPE [43]. Thus, GmNAC081 emerges as a positive regulator of naturally programmed leaf senescence, which in turn may be integrated with multiple-stress-induced PCD via the NRPs/NACs/VPE regulatory circuit.

As a PCD-mediated phenomenon, senescence may provide a stress-escape mechanism. However, the regulatory signaling modules that integrate natural and stress-induced senescence very often inversely modulate senescence and stress tolerance. In the case of the NRPs/GmNACs/VPE signaling module, overexpression of GmNAC081 has been shown to accelerate leaf senescence and decrease drought tolerance considerably [41,43]. In the GmNAC081-induced transcriptome, SAGs and cell death inducers predominate in the upregulated changes, whereas regulators of ABA signaling are predominantly downregulated. This transcriptional landscape derived from GmNAC081 overexpression further supports the argument that leaf senescence is molecularly linked to drought tolerance. Compelling evidence in the literature has linked drought tolerance with the impairment of stressed leaves’ ability to undergo senescence [126]. Further supporting this interpretation, the overexpression of a negative modulator of the DCD/NRP-mediated cell death signaling, the molecular chaperone binding protein (BiP), has been shown to delay leaf senescence and enhance drought tolerance [75,127].

The binding protein (BiP) belongs to the HSP70 family and plays a crucial role in the unfolded protein response (UPR) pathway. The chaperone BiP is an ER-resident protein that acts as a sensor of changes in ER homeostasis and accumulation of unfolded proteins in the organelle lumen [128,129]. Furthermore, BiP has been shown to attenuate ER-stress- and osmotic-stress-mediated cell death by downregulating GmNRP-A, GmNRP-B, and GmNAC081 expression under stress conditions. In contrast, the silencing of endogenous BiP enhances the stress-induced cell death response [109]. Therefore, the BiP cytoprotective function is associated with the inhibition of the DCD/NRP-mediated cell death response. As multiple stresses trigger DCD/NRP-mediated cell death signaling, the engineered control of this pathway might permit the coordination of adaptive cellular responses under a large array of stress conditions.

## 4. Conclusions

The NAC proteins comprise a large family of plant-specific transcription factors involved in stress responses and development. In general, approximately 40% of the NAC genes are upregulated in leaf senescence in flowering plants. Most of the knowledge about NAC-SAGs is derived from Arabidopsis studies and may not apply to all flowering plants. However, the high conservation among NACs from different crops and plant model systems suggests that biological information regarding Arabidopsis NAC-SAGs may be transferable to the studies of crops. In soybean, among 54 GmNAC-SAGs identified by functional genomic approaches, only three of them, GmNAC030, GmNAC065, and GmNAC081, have been functionally characterized. Interestingly, the functional characterization of GmNAC065 demonstrated that it plays a similar function as its Arabidopsis ortholog AtNAC083 (VNI2). Both are negative regulators of leaf senescence and control similar regulatory networks, targeting similar SAGs. These findings further substantiate the notion that similarity-based clustering of the NAC superfamily members along with expression profile correlates with their function, providing a reliable means to rationalize functional studies of the NAC gene family. GmNAC065 expression is correlated with drought tolerance, as it is highly expressed in drought-tolerant genotypes. These findings may implicate GmNAC065 as a potential target for molecular breeding and engineering towards drought tolerance.

GmNAC030 and GmNAC081 were first identified and characterized in soybean, but their orthologs from Arabidopsis show similar conserved functions. Both GmNACs are positive regulators of leaf senescence and are downstream components of a signaling pathway that integrates multiple environmental signals into the plant’s internal senescence information, culminating in PCD. Many aspects of this cell death pathway have been elucidated in the last years. We now know that multiple stress conditions activate the upstream component NRP, which is controlled by the ERD15 TF. NRPs induce GmNAC030 and GmNAC081, which form a dimer to fully induce the expression of VPE, the executioner of the cell death program via vacuole collapse. GmNAC081 and GmNAC030 also target the promoter of other senescence-promoting hydrolytic enzymes and repress the expression of senescence suppressors. However, several crucial players of this pathway are missing, and relevant questions remained unanswered. For example, we still do not know about the most upstream component of the pathway that senses external and internal signals of leaf senescence. How the signal is relayed from NRPs to GmNACs is unclear. We know, however, that modulation of this cell death pathway may confer tolerance to drought and other abiotic stresses. In fact, the protective function of BiP against water dehydration has been associated with its capacity to negatively modulate the extent of the cell death response resulting from the activation of DCD/NRP-mediated signaling. These findings might implicate this pathway as an excellent target for engineering superior crops.

## Data Availability

Not applicable.
